# Public sector nurses in Swaziland: can the downturn be reversed?

**DOI:** 10.1186/1478-4491-4-13

**Published:** 2006-05-31

**Authors:** Katharina Kober, Wim Van Damme

**Affiliations:** 1 Department of Public Health, Antwerp Institute of Tropical Medicine, Antwerp, Belgium

## Abstract

**Background:**

The lack of human resources for health (HRH) is increasingly being recognized as a major bottleneck to scaling up antiretroviral treatment (ART), particularly in sub-Saharan Africa, whose societies and health systems are hardest hit by HIV/AIDS. In this case study of Swaziland, we describe the current HRH situation in the public sector. We identify major factors that contribute to the crisis, describe policy initiatives to tackle it and base on these a number of projections for the future. Finally, we suggest some areas for further research that may contribute to tackling the HRH crisis in Swaziland.

**Methods:**

We visited Swaziland twice within 18 months in order to capture the HRH situation as well as the responses to it in 2004 and in 2005. Using semi-structured interviews with key informants and group interviews, we obtained qualitative and quantitative data on the HRH situation in the public and mission health sectors. We complemented this with an analysis of primary documents and a review of the available relevant reports and studies.

**Results:**

The public health sector in Swaziland faces a serious shortage of health workers: 44% of posts for physicians, 19% of posts for nurses and 17% of nursing assistant posts were unfilled in 2004. We identified emigration and attrition due to HIV/AIDS as major factors depleting the health workforce. The annual training output of only 80 new nurses is not sufficient to compensate for these losses, and based on the situation in 2004 we estimated that the nursing workforce in the public sector would have been reduced by more than 40% by 2010. In 2005 we found that new initiatives by the Swazi government, such as the scale-up of ART, the introduction of retention measures to decrease emigration and the influx of foreign nurses could have the potential to improve the situation. A combination of such measures, together with the planned increase in the training capacity of the country's nursing schools, could even reverse the trend of a diminishing health workforce.

**Conclusion:**

Emigration and attrition due to HIV/AIDS are undermining the health workforce in the public sector of Swaziland. Short-term and long-term measures for overcoming this HRH crisis have been initiated by the Swazi government and must be further supported and increased. Scaling up antiretroviral treatment (ART) and making it accessible and acceptable for the health workforce is of paramount importance for halting the attrition due to HIV/AIDS. To this end, we also recommend exploring ways to make ART delivery less labour-intensive. The production of nurses and nursing assistants must be urgently increased. Although the migration of HRH is a global issue requiring solutions at various levels, innovative in-country strategies for retaining staff must be further explored in order to stem as much as possible the emigration from Swaziland.

## Background

For a long time a rather neglected resource of health systems, the health workforce – or human resources for health (HRH) – has recently been receiving increased attention from the international health community. The HRH shortage is now being identified as one of the major challenges for improving health in low-income countries. The Joint Learning Initiative estimates that: "sub-Saharan countries must nearly triple their current numbers of workers by adding the equivalent of one million workers [...] if they are to come close to approaching the Millennium Development Goals for health" [1].

HIV/AIDS has increased the burden on existing health facilities and is increasingly becoming a major direct and indirect cause for health worker attrition. In many countries of sub-Saharan Africa, people with HIV-related illnesses occupy more than 50% of hospital beds and there is abundant evidence that health workers are overwhelmed by the demand for care [2,3]. At the same time, the lack of health workers in sub-Saharan Africa is regarded by many as the key bottleneck for scaling up antiretroviral treatment (ART) for the millions in need of it [2,4,5].

Swaziland, a small, lower middle-income country of just over one million inhabitants, is, like most other countries in southern Africa, facing a HRH crisis that is exacerbated by the impact of HIV/AIDS. With an adult HIV prevalence of approximately 42% in 2004, Swaziland is reckoned to have had around 220 000 people living with HIV/AIDS in 2003 and more than 36 000 requiring ART by the end of 2005 [6]. Swaziland's *Health sector response to HIV/AIDS plan 2003–2005 *identifies human resource shortages at all levels of the health sector as a serious constraint for scaling up ART. It identifies the problem of brain drain and the impact of HIV/AIDS as major factors contributing to the dire situation and calls for urgent measures to be taken to tackle the HRH crisis [7].

The present country case study of HRH is a more in-depth follow-up of the authors' previous exploration, in January 2004, of the impact of HIV/AIDS on health systems and the main issues related to scaling up ART in four countries in southern Africa [4]. Having identified the lack of HRH as the main bottleneck for scaling up ART in the region, we conducted a two-week rapid assessment study in Swaziland in June 2004 to describe and analyse the HRH situation, to make projections for the future based on the available data and to identify major factors contributing to the HRH bottlenecks for scaling up ART in Swaziland. We updated the 2004 study 18 months later, in December 2005, in order to illustrate the responses and their effects on the HRH situation in the country.

## Methods

Our rapid-assessment study comprised two visits to Swaziland. During the first two weeks in June 2004 we collected both quantitative and qualitative data.

However, with no information system readily available in Swaziland that integrates information about numbers, deployment, educational levels and attrition of the health workforce, much of our quantitative information is from secondary sources, which probably results in a number of inaccuracies.

The follow-up visit, in December 2005, served the main purpose of finding out what steps had been taken to deal with the HRH situation. Due to the mentioned limitations of the quantitative data from 2004, we relied mainly on qualitative methods for the second purpose, instead of trying to quantify minor staff changes with the help of incomplete documents.

**Figure 1:**
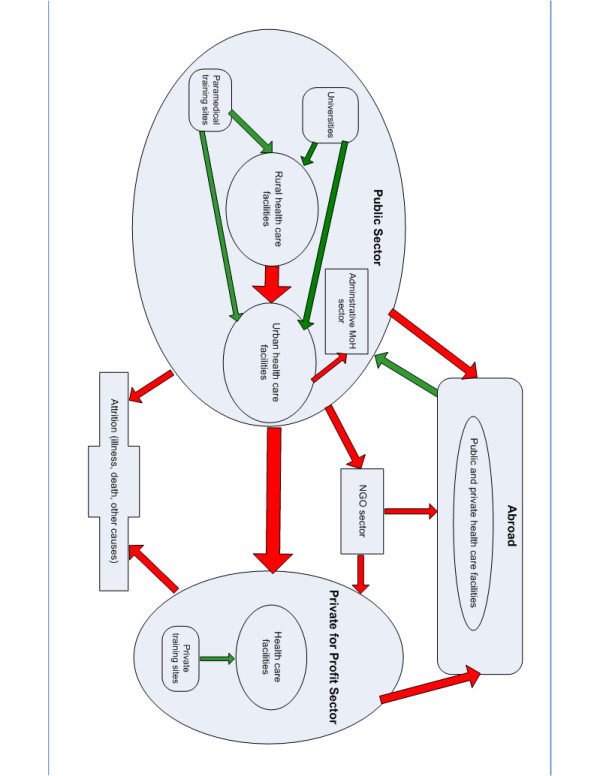
Map of HRH flows in Swaziland.

The HRH movements are described in a flow diagram (Figure 1), yet the absence of a comprehensive HRH information system did not allow an accurate quantification of the flow of health workers, whether between regions or sectors or internationally. Since it was not possible to obtain reliable data on employment in the private, for-profit sector, we focused our analysis on the public and mission sectors. We looked at three categories of staff – physicians, nurses and nursing assistants – and distinguished two inventory levels: current post establishment and actual employment. No further distinction was made between professional specializations within the three staff categories.

In order to obtain our data, we analysed a variety of primary sources, such as staff establishments and lists of medical students, as well as secondary sources, such as reports and studies of the HRH situation in the country [8]. We triangulated the written data by conducting semi-structured interviews with key informants from the Ministry of Health and Social Welfare (MOHSW) and the National Emergency Response Council on HIV/AIDS (NERCHA) and group interviews with management staff of health facilities, nursing schools and the nursing association.

Where no hard data were available, we worked with estimates based on this variety of sources. Thus, findings on the attrition of nurses are based on the information obtained during our interviews and discussions and on an extrapolation of population data to the health workforce. Since the results of an HIV/AIDS impact study on the health workforce in Swaziland, conducted in 2005, were not yet published at the time of our study, we based our attrition estimate on findings from other impact studies in a number of sub-Saharan countries, such as Botswana, Mozambique, South Africa and Zambia. The HIV prevalence and AIDS-related mortality among nurses in these countries have been estimated to be at least as high as in the general population [9-11]. We therefore supposed that the HIV prevalence among nurses in Swaziland is similar to the 42% among the general adult population recorded in the national 2004 antenatal clinic survey [12]. In the absence of ART, we assumed an approximate 10% annual mortality rate among the HIV-infected population. We used these fractions to project an attrition scenario among the nursing workforce.

Due to our rapid assessment approach, we could neither visit all health facilities nor interview all stakeholders, a fact that may have introduced bias in the sample visited and in the key informants selected.

## Results

### The health care system, HRH, HIV/AIDS and ART scale-up: general findings in 2004 and 2005

Swaziland's health care system comprises public, private not-for-profit, private for-profit and industry-owned facilities. The majority of the private not-for-profit facilities are owned by missions but receive most of their subsidies from the Swazi government. The public and mission sectors operate six hospitals, eight public health units and five health centres offering both preventive and curative services, with between them a total of 1851 beds. Community-based care is offered in these sectors by 89 health clinics and 174 outreach clinics. In the private for-profit sector there are more than 50 clinics with and without beds; various industries own between them around 40 facilities, ranging from small health posts to clinics with more than 35 beds.

As Swaziland has no medical school, students must go abroad to obtain a medical degree. Nurses are trained at two sites in the country. One school is located within the compound of a mission hospital; the other was upgraded in 1997 to be part of the University of Swaziland. Basic nursing training lasts three years, but the majority of students do either one additional year for midwifery or a five-year course to obtain a Bachelor in Nursing Sciences degree. Some 125 registered nurses have been trained for a total of six years to become Family Nurse Practitioners. There is one school for nursing assistants in the country, where the training lasts two years.

At the time of our two visits, several ministries were involved in HRH-related decision-making. Overall responsibility for policy, management and planning for HRH in the public services lay with the Ministry of Public Works and Information. The Civil Service Board was in charge of technical recruitment matters; the Ministry of Education dealt with the pre-service training of all health workers. The MOHSW was responsible for delivering health services. The four Regional Management Committees of the MOHSW and the regional hospitals were supposed to submit their staffing plans to the central level of the MOHSW, which in turn made a request to the Civil Service Board.

Referring to a study commissioned by the MOHSW in 2000, WHO gives the total number of health workers in Swaziland as 3726, spread over 17 professional groups [8]. At the time of our visit in 2004, public and mission health facilities had 1481 posts and the actual number of staff reported to us as currently working in these facilities was 1184. The zero growth in the public sector over the past few years meant that spending on HRH could not be increased [8] despite the shortage of health workers confirmed by the Personnel Officer at the MOHSW. He reported requesting 200 additional health workers for the fiscal year 2003/2004 and being granted only two new medical staff. According to several informants from public and mission hospitals, the number of new posts has not increased since 1985, despite an increased burden of care.

The impact of HIV/AIDS on the health sector is most visible in the hospitals, where it is estimated that 80% of bed occupancy in the medical and paediatric wards is HIV/AIDS-related. Doctors from a mission hospital estimated that five years ago they would spend an average of five minutes per patient on a ward round, while presently this was more likely to be 20 to 30 minutes. This was seen as a consequence of the increasing number of terminally ill patients needing time-intensive care. There is an increased demand for health services, and health workers speak of feeling overwhelmed and burnt-out.

The government of Swaziland committed itself to providing ART to 12 000 people by the end of 2005, while WHO's "3 by 5" target would have been 16 000 [13]. The provision of ART in the public sector started in late 2001 in Mbabane hospital, and free-of-charge ART has been offered since November 2003. By June 2004 the programme had been extended to five public (government and mission) hospitals treating a total of nearly 3200 people. At the same time, two facilities in the private for-profit and commercial sectors were providing ART to more than 700 people.

By December 2005, ART was provided by all six public hospitals in the country, by five public health centres and by six facilities in the private sector. We were informed by the director of the Swazi National AIDS Programme that close to 12 000 people were alive and on ART by December 2005. According to the *Second national multisectoral HIV and AIDS strategic plan 2006 – 2008*, the government is planning to increase this number by approximately 13 000 new ART patients per year. The plan also states that staff shortages seriously compromise the effective delivery of ART to those in need. It estimates that 410 nurses and 247 nursing assistants were needed to support ART services in 2005 [6].

### Physicians and nurses: findings and estimates in 2004

#### Numbers and distribution

##### Physicians

The doctor:population ratio, placed by WHO at 17.6 per 100 000, is based on the total of 182 doctors who were registered with the medical and dental council in Swaziland in 2004 [14]. The public and mission sectors have 90 posts, of which 50 were filled in June that year (Table 1). Of the 92 remaining registered doctors, 80 were working in the private for-profit sector and 12 in the industry-owned sector. More than 70% of doctors in the government and mission sector were of non-Swazi nationality. For the private sector this figure was estimated at 60%. In the public sector, two urban hospitals with a total of 844 beds employed 34 doctors, while the remaining four rural hospitals, with 826 beds, employed 16 doctors.

**Table 1:** HRH inventory of the public and mission health sectors in Swaziland, June 2004

	**Total registered (per 100 000 population)**	**Official posts in public sector**	**Actually working in public facilities (per 100 000 population)**	**Shortfall (in %)**
Physicians	182 (17)	90	50 (5)	40 (44%)
Nurses	3261 (296)	944	758 (69)	183 (19%)
Nursing Assistants	700 (63)	454	376 (34)	78 (17%)

**Total**	**4143**	**1488**	**1184**	**301**

##### Nurses and nursing assistants

In 2003 the nursing council had registered 3261 nurses, which translates into a nurse/population ratio of 296 per 100 000. Some 758 of the 944 established nursing posts in the public and mission sectors were filled by June 2004. The nurse/population ratio in the public and mission sectors is therefore around 70 per 100 000. The official number of nursing posts was widely regarded as inadequate for the actual workload in the health facilities.

The chief nursing officer at the MoH told us in December 2005 that often nursing staff is seconded from the hospitals to specific programmes or research projects. This practice makes it look as if all posts in the establishment were filled, while in fact the hospital wards are short of staff. In 2004 mission hospitals were generally better staffed, apparently because of their ability to employ non-Swazi nurses, a strategy that was not permitted in the government sector until July 2004. Due to the lack of data for the private sector, we could not clarify which proportion of the remaining 2317 registered nurses are employed in the private and commercial sectors. At the biggest industry-owned health centre we visited in 2004, the staffing ratio was more favourable than in the public sector, with 34 nurses, 3 doctors and 10 nursing assistants being responsible for 37 beds.

The total number of registered nursing assistants could not be established, but was estimated by the Swazi nursing association at around 700. Of 454 established posts in the public and mission sectors, 376 could be confirmed as filled in 2004 (Table 1).

#### Inflow, outflow and attrition

##### Physicians

Most medical students go to South Africa, where the majority study at Medunsa University. In June 2004, a total of 72 students were following medical studies abroad; there are around 15 graduates each year. Students receive a government bursary which, theoretically, they are to pay back in the years after graduation. According to respondents from the MHOSW, only a minority of graduates return to Swaziland, and even fewer enter the public sector.

The outflow of Swazi doctors from the public service has long been recognized as problematic, as is shown by a 1996 report requested by the Parliament and submitted to the Principal Secretary of the MOHSW: *Report on why medical doctors leave government employment for work without the Swazi public service *[15]. Its findings include issues such as a lack of proper coordination and planning in the recruitment system of newly trained doctors, perceived preferential treatment of foreign doctors to Swazi doctors by the MOHSW and an inadequate career structure. Several of the physicians we interviewed described these issues as being still unresolved.

##### Nurses and nursing assistants

The two nursing schools receive between them around 500 annual applications; they are able to take 80 to 90 entrants. The school for nursing assistants has an annual output of 20 to 30. In June 2004 the directors and lecturers at both schools told us they were already working to full capacity and could not take more students. Insufficient student bursaries from the MOHSW, lack of teaching staff, lack of accommodation and insufficient possibilities for the practical stages of the training were given as the main reasons. According to the directors of the training sites, usually all students are recruited into public service soon after their graduation.

Hospital managers found it difficult to estimate the number of nurses who died or left the service because of terminal illness in 2003 and 2004. However, our respondents from the hospitals told us about high absentee rates among the staff because of their own illness, care commitments in the family or funerals. In one hospital it was reckoned that on average only half of all nurses could be counted on to be able to do their full duty at any one time. In the smallest hospital, with 28 nursing posts, seven nurses were thought to have died between January 2002 and June 2004. Management staff from the government hospital in Mbabane, with 110 filled nursing posts, estimated that each year three or four nurses had died over the past couple of years.

The most striking recent development observed by our informants in 2004 was clearly the extent of the emigration of nurses from Swaziland. The two major hospitals, with 203 and 125 established nursing posts, respectively, reckoned that each of them had lost between 25 and 35 nurses in 2003. In the smallest hospital in the country, a mission facility with 28 posts, 10 nurses had come to get their transcripts to apply for a position abroad in the first six months of 2004. The Department of Training at the MOHSW estimated that four to five nurses had been leaving the country weekly in the same period and the nursing association reported of a batch of 27 nurses who left for the United Kingdom in June 2004.

The extent of the nurses' desire to find work abroad is illustrated by the finding that there were at least four private personnel recruitment offices in Swaziland in June 2004. The manager of one of them told us that his office alone had recruited more than 30 nurses to the United Kingdom in the first two months after starting in the business. A nurse tutor, who had asked her students about the motivation for their career choice, told us that more than 50% of first-year students hoped it would give them the opportunity to go abroad.

#### Projections 2004 to 2010

Swaziland's approximate 38% adult HIV prevalence made it possible to estimate that 288 of the 758 nurses employed in the public and mission sectors in 2004 could have been HIV-positive and that 29, or 10% of all HIV-positive nurses, might have died. We could therefore project that around 3% to 4% of the entire nursing workforce would die from AIDS annually. In the cases of the above hospitals with 203, 125 and 28 established nursing posts, this would have meant that 77, 48 and 11 nurses, respectively, might have been HIV-positive in 2004, and that the hospitals would lose a minimum of 8, 5 and 1 nurses each in that year. The impact of HIV/AIDS on the entire public health workforce would be considerable, with 20 to 30 deaths of nurses annually over the next five years.

This yearly loss of 3% to 4% of the public health workforce due to AIDS is alarming. Still, in the perception of our respondents the attrition of the health workforce due to HIV/AIDS seemed to pale in comparison with the exodus of nurses, which was believed to have grown to this massive scale only over the course of 2003. Hospital managers found it difficult to estimate the number of nurses who had died over the same period of time. Without doubt, emigration was a huge issue in 2004, and based on the information from our informants we made a conservative estimate that 100 nurses were leaving Swaziland every year.

Our 2004 projections show that both emigration and attrition due to HIV/AIDS pose a serious double threat for Swaziland's health system, which, if not effectively tackled, would mean the loss of more than 330, or 44%, of the nursing workforce in the public sector up to 2010 (Table 2).

**Table 2:** Projections of the nursing workforce in the public and mission health sectors until 2010, based on findings in 2004

	**Mid-2004**	**Mid-2005**	**Mid-2006**	**Mid-2007**	**Mid-2008**	**Mid-2009**	**Mid-2010**
Total nurses in the public and mission sectors	758	694	634	577	524	473	426
Total inflow	80	80	80	80	80	80	80
Training output	80	80	80	80	80	80	80
Immigration	0	0	0	0	0	0	0
Total outflow	144	140	137	133	130	127	125
Normal retirement*	15	14	13	12	10	9	9
Retirement/death due to AIDS**	29	26	24	22	20	18	16
Emigration	100	100	100	100	100	100	100
Net loss per year	64	60	57	53	50	47	45

* Rate calculated at 2%

** Based on 38% HIV prevalence and estimating that 10% of HIV+ people have AIDS

### HRH: findings and estimates, 2005

#### Numbers and distribution

During our second visit in December 2005, we could not obtain an updated list of filled posts of either doctors or nurses in the public sector. The total number of doctors registered with the medical and dental council had fallen from 182 to 167. In 2004 as well as in 2005, informants from the health facilities and the central MoH criticized the establishment, saying that its adjustment to the increased workload was overdue.

#### HRH policy initiatives between 2004 and 2005

By June 2004, the shortage of health workers was acknowledged by all stakeholders in Swaziland as a serious problem, compromising not only the ART scale-up but also the quality of the general health services. However, the absence of a HRH monitoring system made it difficult to quantify the problem, let alone plan an adequate response based on anticipated developments in the HRH sector. Therefore, the development and subsequent implementation of a HRH policy and strategic plan, as initiated by the MoH with technical support from WHO, are important steps in response to the health workforce crisis. According to WHO, the HRH policy foresees an improved integration of HRH planning activities both between ministries and between the regional health management teams and the central level of the MoH.

According to information from WHO in December 2005, the government is also planning to simplify the current HRH policy and planning structures. The aim is to establish one body, the Health and Social Welfare Service Commission (HSWSC), that will be responsible for the recruitment, deployment, development, motivation, retention and discipline of all health care staff.

#### Policy initiatives and their effects to increase the inflow

##### Influx of foreign staff

The lack of medical doctors in the public health system, particularly in view of the needs for the ART scale-up, has prompted NERCHA to include a request for 19 general doctors and 4 paediatric specialists in Swaziland's proposals to the Global Fund to Fight AIDS, TB and Malaria (GFATM) in rounds two and five. According to NERCHA, nine foreign doctors could be recruited with Global Fund money up to December 2005. The MoH has also negotiated with the Ministry of Public Works to lift the ban on the recruitment of foreign nurses into government service in July 2004. Between then and December 2005, 32 additional foreign nurses could be recruited for the government health sector with funds from the GFATM. This measure can of course be criticized on the grounds that, while the recruitment of foreign staff helps overcome staff shortages in Swaziland within a relatively short time, it aggravates the HRH situation in the neighbouring countries.

##### Increased training output

With regard to nursing training, WHO informed us of MoH plans to assess pre-service training needs and requirements, taking into account the high attrition rate among health workers. At the Faculty of Health Sciences we were told that they intend to double their intake of students and were waiting for MoH approval of their plans to expand the infrastructure and increase the number of tutors accordingly. According to the deputy nursing officer at the MoH, increasing the number of tutors or their salaries at the faculty is problematic because they are not employed by the MoH but by the faculty itself.

#### Policy initiatives and their effects to decrease emigration

##### Retention strategies

In order to counterbalance the losses due to emigration, the MoH has started to develop new retention strategies. In April 2005, the government raised the salaries of the civil servants by around 60%. Our interviews in December 2005 show that the salary rise may indeed help to keep nurses and doctors in the public sector, possibly even draw some back from the private for-profit sector. According to the MoH, there are also plans to improve retention with non-monetary motivation strategies for all health staff, such as better accommodation, child care facilities and easier access to car and housing loans for nurses.

Informants from the MoH and the Nursing Association told us that the salary increase in the government sector had substantially changed the situation of the internal market for health workers. Apparently it has become more attractive for nurses to work in the public sector instead of the private sector. The personnel manager of a private clinic in Mbabane confirmed this by saying the clinic could not afford salaries as high as the increased ones in the public sector, nor could it provide additional loan schemes. The only chance of keeping its nurses was to offer better working conditions than the public sector. According to the director of the Swazi Nursing Association, the private sector is now facing double competition: from abroad and from the higher salaries in the public service.

However, these observations did not conform to the comments from the doctors and nurses we talked with in the government hospitals. While people appreciated the salary increase, the main source of dissatisfaction in 2004 and 2005 remained the working conditions in the public sector. According to the nursing association, 21 nurses left Mbabane Hospital in the month of the salary increase.

Doctors and nurses felt overworked and complained to us about the inadequate number of nurses, the lack of essential material and equipment and the poor condition of the hospital infrastructure. Several doctors voiced their frustration about not being able to provide medical care of high quality under the existing conditions. Some also mentioned the lack of medical specialists in the country as unsatisfactory for a conscientious medical professional who sees many patients who would need to be referred. Nurses also mentioned again in 2005 their fear of infection with HIV because of the insufficient and poor-quality material and equipment in the public hospitals. We could not find out whether the retention plans of the MoH also included an improvement of the general working conditions in the public sector.

Still, in December 2005 the deputy director of nursing at the MoH provided us with a list of nurses who had left the government service since 2003, based on figures from the emigration office. The data in this list are neither complete nor 100% accurate, yet they may give an approximation of the real extent of emigration, which somehow differs from the estimations made by our informants. According to the emigration office, 91 nurses left government service in the 22 months between March 2003 and October 2005. Of the 84 who went abroad, 65 went to the United Kingdom and 19 destinations remain unconfirmed. The remaining seven left for various destinations within the country (private, retirement). Of the 65 nurses who departed for the United Kingdom, only five did so in 2005. All 19 nurses with unconfirmed destinations left before 2005.

These official numbers do not match those we obtained from the Director of the Swazi Nursing Association, according to whom the number of nurses leaving the country in 2005 was around six per month: roughly half the number in 2004. This estimation is based on the number of nurses who approach the Nursing Association for an official letter they are required to show to the emigration office. These figures, while leaving a large margin of uncertainty, point to a possible decrease in emigration, which could be an early effect of the retention measures introduced by the government.

##### ART scale-up to reduce attrition

There are currently no official estimates of the attrition rates due to illness or death in the public health sector. In December 2005 we were informed that an HIV/AIDS impact study had been conducted but that the draft version with the findings was not yet ready for distribution.

Regarding nurses' access to ART, it is possible that the proportion of nurses on treatment equals the proportion among the general HIV positive population on treatment. Yet, we learnt from our interviews that there is a specific Swazi dimension to the problem of health workers' access to ART.

As the country is very small, there is very little chance of anonymity in general, but even less so for health workers. The short distances and good infrastructure make it possible for most people to travel with relative ease and to receive ART in facilities away from home. Health workers, however, are quite a small professional group, in which it is hardly possible to obtain ART anonymously in any facility. Therefore it can be assumed that the proportion of nurses on ART lies below the proportion among the general population in need of ART. Still, it is very likely that the health workforce has also benefited from the progress of the general ART scale-up, with one third of the people in need of ART on treatment by the end of 2005.

#### Projections 2005 to 2010

We use the described HRH policy initiatives as variables for establishing a number of future projections different from those based on our findings from 2004.

##### Variable 1. ART scale-up

Starting from the latest estimate of an approximate 42% adult HIV prevalence, we calculate that 318 of the 758 nurses employed in the public and mission sectors may currently be HIV-positive. Assuming that the proportion of nurses on ART is roughly equivalent to the proportion of the general HIV-positive population on ART, around one third of those in need of ART would receive it. If the ART scale-up is maintained at that rate (i.e. one third of those in need annually on ART), with every other variable remaining unchanged, the loss of nurses up to 2010 would fall to 300 nurses, or 40% of the entire public nursing workforce (Table 3).

**Table 3:** Projections of the nursing workforce in the public and mission health sectors in 2010, based on findings in 2005

	Scenario mid-2010 based on 2004 estimates*	**ART scale-up**	**...plus reduced emigration**	**...plus increased foreign recruitment**	**...plus doubled training output**	**All measures combined**
Total nurses in the public and mission sectors by mid 2010	*426*	474	734	618	538	974
Total inflow	*560*	480	480	660	560	740
Training output	*560*	480	480	480	560	560
Immigration	*0*	0	0	180	0	180
Total outflow	*937*	764	504	800	780	524
Normal retirement¶	*82*	77	87	84	75	95
Retirement/death due to AIDS§	*155*	87	122	117	105	134
Emigration	*700*	600	295	600	600	295
Total loss between 2004 and 2010	*377*	284	24	140	220	-216

* Column taken from Table 2 (under the 2004 assumption that no HRH measures are taken)

¶ Rate calculated at 2%

§ Based on the assumption of 42% HIV prevalence; 10% of HIV+ people have AIDS and need ART; one third of those in need are put on ART (i.e. the current rate of ART scale-up)

##### Variable 2. ART scale-up plus reduced emigration

If we accept the view of our Swazi informants that emigration had reached its peak in 2004 and has fallen since, we take the mean of the lowest (46) and highest (144) estimates of annual emigration before 2005 provided by the emigration office and the Nursing Association, respectively, and arrive at 95 nurses who left the country in 2004. The mean from the same sources for 2005 would be 39 nurses. Assuming that emigration will continue at around 40 nurses per year, our projection for 2010 shows a loss of 24, or 3%, of the total public nursing workforce (Table 3).

##### Variable 3. ART scale-up plus import of foreign nurses

Having lifted the recruitment ban for foreign nurses, Swaziland could consider employing an increasing number of nurses from other African countries. Until now, the recruitment of foreign nurses seems to have been a one-off initiative financed with Global Fund money. Continuing with the employment of around 30 foreign nurses annually (as in 2004/2005), the total workforce would have lost 140, or 19%, of its nurses by 2010 (Table 3).

##### Variable 4. ART scale-up plus increased training capacity

If the MoH can manage to double the training capacity of the nursing training facilities by mid-2006, the results in terms of nursing graduates cannot be expected before 2009. Thus the impact of this measure would be felt only in the last two years of our projections but would still reduce the loss to 220, or 29%, of nurses until 2010 (Table 3). However, in the long run a doubled training output would slowly reverse the trend of annual losses, as from 2009 onwards the total annual inflow would exceed the annual outflow.

##### Variable 5. All measures combined

If the HRH policy in Swaziland combined all the measures discussed above, this would result in quite a spectacular reverse of the HRH depletion scenario as projected based on our observations in 2004. In 2010, the current baseline of 758 nurses would have increased by 216 nurses, or 28% (Table 3).

## Conclusion

### Summary of findings

Our findings from June 2004 showed that the public health sector in Swaziland was losing its health workers at an alarming rate. Emigration and attrition due to HIV/AIDS were the major causes of these losses. Without new HRH policy initiatives it looked as if the public health sector could lose up to 44% of its entire nursing workforce by 2010. During our second visit, in 2005, we realized that measures had indeed been taken to overcome the HRH crisis, and we could observe some possible early effects of such measures that allowed us to develop more optimistic future scenarios.

Each of the four HRH policy measures being discussed or already implemented by the government of Swaziland would slightly reduce the losses of the health workforce. The reduction of emigration is the measure with potentially the biggest impact. However, it is the combination of all measures in a comprehensive HRH policy that would result in a spectacular reversal of the trend and lead to growth of the health workforce in Swaziland.

### Limitations of findings

The absence of a comprehensive HRH information system in Swaziland made it necessary to work with estimates based on a variety of sometimes-contradictory sources. Therefore our projections depart from baseline calculations that may themselves provide a slightly inaccurate quantification of the actual situation of the health workforce in Swaziland.

We are also aware that our projections may not capture all potential effects of HRH developments and policies, nor do they include a quantification of the workload and possible future changes to it. Still, they provide us with a rough but very illustrative idea of future developments that may result from present actions taken or not taken.

Our rapid assessment approach did not allow us to visit all health facilities or interview all stakeholders. This may have introduced bias in the sample visited and in the key informants selected.

### Implications of findings

We argue that the trend of a diminishing nursing workforce in Swaziland can be reversed if as many as possible of the planned policy initiatives are swiftly and jointly implemented.

We would further stress the particular importance of implementing as soon as possible the planned extension of training capacity of the country's nursing schools. Depending on the scale of the extension, such an initiative could substantially increase the number of nurses available for public health service in Swaziland in the medium and long term. With more than 500 nursing applicants per year, the lack of accommodation, grants and tutors should be overcome as obstacles for accepting more students into nursing school. Yet, training more nurses alone may not necessarily improve the HRH situation in the Swazi health system as long as so many of the new students make their career choice with the intention of working abroad.

Therefore, there is a need for qualitative research into the right mix of retention measures, beyond financial incentives such as salary increases. To our knowledge, no comprehensive job satisfaction survey has been conducted so far in the Swazi health sector. Some retention measures, such as easier access to different kinds of loans, seem to be much appreciated by nurses, but other strategies being discussed at the MoH, such as child care facilities and subsidized housing, should also be further investigated and implemented as swiftly as possible if deemed useful.

Yet, in our interviews with nurses and doctors, the actual working conditions in the public sector figured more prominently than the issue of salaries and benefit packages. The importance of good working conditions is illustrated by the example of a private clinic in Mbabane with 32 beds and 20 full-time nurses. This clinic pays the same salary as the government and does not offer any extra loan or pension schemes, yet has not lost any of its staff to emigration in the past couple of years. The clinic's personnel manager attributes this to some features that distinguish the clinic from public sector hospitals, such as a lower workload, a different shift system, many training opportunities and very close tutoring and guidance on good care practices.

While we assume that the ART scale-up in Swaziland is also reducing attrition of the health workforce due to HIV/AIDS, there seem still to be problems of access due to the perceived lack of anonymity for health workers when seeking treatment. While the solution will lie ultimately in reducing the stigma around HIV/AIDS, we would recommend exploring other, more intermediate solutions to the problem of health workers' access to ART.

The lack of HRH in Swaziland is widely regarded as the main bottleneck to scaling up ART and maintaining thousands of patients on ART over time. Therefore we would also recommend intensified research into the potential of making the current ART delivery model less HRH-intensive. Several studies in other sub-Saharan African countries with severe HRH shortages have drawn attention to the need for context-specific ART delivery models that require considerably less time from doctors and nurses [2,5,16].

Some of our respondents at the MoH and NERCHA regard the stronger involvement of PLWHA as crucial for the successful scale-up of ART in Swaziland. Yet up to now PLWHA only assist with logistics and patient flow in a few ART clinics, an activity for which they are paid a small incentive from Global Fund money. A 2004 directory lists almost 50 PLWHA associations in Swaziland [17] and we know that more than 11 000 people are on ART. We would therefore suggest exploring in more depth the potential and the capacity-strengthening needs of these groups and people on treatment and identify areas for the involvement of PLHA beyond mere support functions but for tasks up to now reserved for doctors and nurses [18,19].

However, while increasing the production of health workers, improving the retention strategies, fighting the stigma of AIDS and adjusting ART delivery models are necessary and important measures for dealing with the HRH crisis, emigration of HRH remains a complex issue that cannot be tackled at the national level alone [20,21]. It is unlikely that the problem of emigration can be tackled in the long term without addressing the structural and political factors that affect HRH at national and global levels [20,22-24].

## Competing interests

The author(s) declare that they have no competing interests.

## Authors' contributions

Katharina Kober and Wim Van Damme designed the study. KK conducted the interviews in Swaziland, reviewed the available documentation of HRH in Swaziland and wrote the initial draft of this article. Both authors analysed the obtained data and WvD revised the subsequent drafts of this article.
